# Comparison of the sensitivity of different criteria to select lung cancer patients for screening in a cohort of German patients

**DOI:** 10.1002/cam4.5638

**Published:** 2023-01-27

**Authors:** Julia Walter, Diego Kauffmann‐Guerrero, Thomas Muley, Martin Reck, Jan Fuge, Andreas Günther, Raphael W. Majeed, Rajkumar Savai, Ina Koch, Julien Dinkel, Christian Schneider, Karsten Senghas, Sonja Kobinger, Farkhad Manapov, Michael Thomas, Kathrin Kahnert, Hauke Winter, Jürgen Behr, Martin Tammemägi, Amanda Tufman

**Affiliations:** ^1^ Department of Medicine V University Hospital, LMU Munich, Member of the German Center for Lung Research (DZL‐ CPCM) Munich Germany; ^2^ Thoraxklinik University Hospital Heidelberg and National Center for Tumor Diseases (NCT) Heidelberg Germany; ^3^ Translational Lung Research Center (TLRC) Member of the German Center for Lung Research (DZL) Heidelberg Germany; ^4^ LungenClinic Grosshansdorf, ARCN, DZL Großhansdorf Germany; ^5^ BREATH ‐ Biomedical Research in Endstage and Obstructive Lung Disease Hannover Standort des Deutschen Zentrums für Lungenforschung Hannover Germany; ^6^ University of Gießen‐Marburg Lung Center (UGMLC), Justus Liebig University Gießen Gießen Germany; ^7^ Institute for Lung Health (ILH) Justus Liebig University Giessen Germany; ^8^ Department of Thoracic Surgery Asklepios Clinic Gauting, Comprehensive Pneumology Center Munich (CPC‐M), Member of the German Center for Lung Research München Germany; ^9^ Department of Radiology Asklepios Clinic Gauting, Comprehensive Pneumology Center Munich (CPC‐M), Member of the German Center for Lung Research Munich Germany; ^10^ Department of Thoracic Imaging University Hospital, LMU Munich Munich Germany; ^11^ Department of Thoracic Surgery University Hospital, LMU Munich, Member of the German Center for Lung Research (DZL‐ CPCM) Munich Germany; ^12^ Department of Radiation Oncology University Hospital, LMU Munich, Member of the German Center for Lung Research (DZL‐ CPCM) Munich Germany; ^13^ Brock University St. Catharines Ontario Canada

**Keywords:** health policy, lung cancer screening, NSCLC, thoracic malignancy

## Abstract

**Introduction:**

Trials of CT‐based screening for lung cancer have shown a mortality advantage for screening in North America and Europe. Before introducing a nationwide lung cancer screening program in Germany, it is important to assess the criteria used in international trials in the German population.

**Methods:**

We used data from 3623 lung cancer patients from the data warehouse of the German Center for Lung Research (DZL). We compared the sensitivity of the following lung cancer screening criteria overall and stratified by age and histology: the National Lung Screening Trial (NLST), the Danish Lung Cancer Screening Trial (DLCST), the 2013 and 2021 US Preventive Services Task Force (USPSTF), and an adapted version of the Prostate, Lung, Colorectal, and Ovarian no race model (adapted PLCOm2012) with 6‐year risk thresholds of 1.0%/6 year and 1.7%/6 year.

**Results:**

Overall, the adapted PLCOm2012 model (1%/6 years), selected the highest proportion of lung cancer patients for screening (72.4%), followed by the 2021 USPSTF (70.0%), the adapted PLCOm2012 (1.7%/6 year) (57.4%), the 2013 USPTF (57.0%), DLCST criteria (48.7%), and the NLST (48.5%). The adapted PLCOm2012 risk model (1.0%/6 year) had the highest sensitivity for all histological types except for small‐cell and large‐cell carcinomas (non‐significant), whereas the 2021 USPTF selected a higher proportion of patients. The sensitivity levels were higher in males than in females.

**Conclusion:**

Using a risk‐based selection score resulted in higher sensitivities compared to criteria using dichotomized age and smoking history. However, gender disparities were apparent in all studied eligibility criteria. In light of increasing lung cancer incidences in women, all selection criteria should be reviewed for ways to close this gender gap, especially when implementing a large‐scale lung cancer screening program.

## Graphical abstract

A comparison of lung cancer screening selection approaches resulted in higher sensitivities in risk‐based models compared to criteria using dichotomized categorical age and smoking history in a German population of lung cancer patients. However, gender disparities were apparent in all studied eligibility criteria. In light of increasing lung cancer incidences in women, all selection criteria should be reviewed regarding ways to close this gender gap, especially when implementing a large‐scale lung cancer screening program.

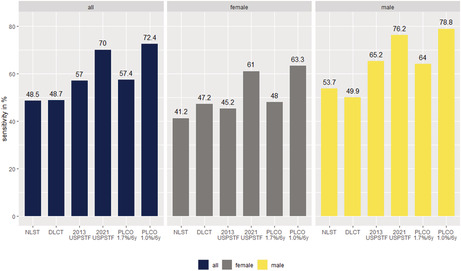

## 1 INTRODUCTION

Trials of computed tomography (CT) lung cancer screening have shown a mortality advantage for patients in both North America and Europe.^1^, ^2^, ^3^ In contrast to some regions of America, Asia, and Europe have not yet widely implemented CT screening. The German S3 Lung Cancer Guidelines were updated in 2018 and now include a “can” recommendation for lung cancer screening using CT Thorax,^4^ meaning that doctors can offer yearly CT screening to patients with a defined risk for lung cancer. Currently, the implementation of a nationwide lung cancer screening program is in planning.

Prior to implementing this screening program, it is important to assess the criteria used in international trials in the German population before using those criteria as the foundation for broad public health measures in this region. Due to regional variability in genetic susceptibility, smoking patterns, and both indoor and outdoor air quality, there may be clinically relevant regional differences in the performance of screening algorithms and risk scores. For this reason, it is important to test screening criteria developed elsewhere in the German population.

Lung cancer screening inclusion criteria can be divided into two categories. First, some eligibility criteria identify high‐risk patients according to age and smoking history, which is easily applicable. Second, lung cancer risk models such as the Bach model, the Liverpool Lung Project model (LLP), or the Prostate, Lung, Colorectal, and Ovarian risk prediction model (PLCOm2012), which additionally take into account factors like personal history of cancer, family history of lung cancer, body mass index (BMI), respiratory comorbidities, and other factors. Though more complex, risk prediction models have been shown to be superior to the dichotomized criteria such as the 2013 and 2021 US Preventive Task Force (USPSTF) at identifying high‐risk patients for screening programs.^5^, ^6^, ^7^, ^8^

Thus far, two studies have analyzed eligibility criteria in the German population. The first study investigated German ever‐smokers in the European Prospective Investigation of Cancer and Nutrition (EPIC) study, who were followed up for 5 years regarding cancer development.^9^ The authors found that all of the established lung cancer risk prediction models performed better at detecting high‐risk patients compared with the simpler eligibility criteria.^10^ Of the risk prediction models, the PLCOm2012 performed slightly better than the LLP and Bach models.^10^ The second study used data from the German Health Update study (GEDA; “Gesundheit in Deutschland aktuell”), a series of cross‐sectional surveys covering health and disease in the German population.^11^ This analysis showed that compared to other criteria the PLCOm2012 had the best concordance between the numbers of lung cancer cases predicted and those reported in registries.^12^ The most recent study aiming at comparing the NELSON and the PLCOm2012 selection criteria the HANSE study (ClinicalTrials.gov Identifier: NCT04913155), started enrolling German at‐risk patients in June 2021. At the time of this study, no results had been published yet.

Although these two studies provide a good basis concerning the performance of selection criteria in a population of healthy patients, further work is required to understand how these criteria perform in a cohort of already diseased patients. Additionally, we need a better understanding of the characteristics (e.g. histology, gender, and molecular pathology) of patients selected and not selected by each of the criteria.

Therefore, in our analysis, we aimed to compare the sensitivity of different lung cancer screening inclusion criteria to select lung cancer patients for screening in a population of German lung cancer patients. We assessed the screening inclusion criteria used in recent large trials including the National Lung Screening Trial NLST/USPSTF,^1^ the Danish Lung Cancer Screening Trial DLCST,^13^ and an adapted version of the PLCOm2012.^5^, ^7^ Additionally, we aimed to test the sensitivity of the selection criteria across different lung cancer histological types and between males and females, as well as to compare other characteristics between the patients who were selected and not selected for screening.

## 2 METHODS

### 2.1 Study design, patient cohort, and data collection

In this retrospective analysis, we used data provided by the data warehouse of the German Center for Lung Research (DZL), covering five major German lung cancer centers consisting of several (university) hospitals and other scientific facilities. The DZL data warehouse provides broad coordinated access to patient‐related lung research data for scientific purposes. Patients included consent to the pseudonymized, pooled use of their clinical data for research purposes. Within the DZL, interdisciplinary teams representing each area of research define basic clinical parameters for the dataset and encourage all contributing sites to include all consenting patients in a prospective manner. The dataset provided by the DZL data warehouse contained 9481 patients with a diagnosis of lung cancer. Variables in the dataset included date of birth, date of diagnosis, gender, histology, smoking status, pack years, height, weight, BMI, documentation of comorbidities such as chronic obstructive pulmonary disease (COPD), and TNM (tumor, nodes, metastases) stage at diagnosis. The depth of documentation varied between datasets. As age and smoking history are the major factors typically used in screening eligibility criteria, we excluded all patients that did not have any information on age and smoking status/pack years.

### 2.2 Ethics statement

Approval for this retrospective non‐interventional study was obtained from the Ethics Committee of the Ludwig‐Maximilians University (reference number 19‐959). This study was conducted in accordance with the Declaration of Helsinki, Good Clinical Practice guidelines, and local ethical and legal requirements.

### 2.3 Selection criteria

We compared the following established lung cancer screening criteria in this analysis: the NLST (inclusion criteria: 55–75 years, ≥30 pack years),^14^ the DLCST (inclusion criteria: 50–70 years, ≥20 pack years),^13^ the 2013 and 2021 USPSTF (inclusion criteria 2013: 55–80 years, ≥30 pack years, inclusion criteria 2021: 50–80 years, ≥20 pack years),^15^ and an adapted PLCOm2012 model (PLCOm2012 with risk thresholds of 1.0%/6 years and 1.7%/6 year).^5^ The risk threshold of 1.7%/6 years has been shown to be comparable to the USPSTF 2013 selection criteria in a North American population,^16^, ^17^ and the 1.0%/6 years threshold is assumed to be comparable to the updated 2021 USPSTF criterion.

### 2.4 Adaptation of variables and handling of missing data

If available, we used variables for the exact date of diagnosis. For variables that may fluctuate over time (e.g. BMI, weight), we used the values closest to the date of diagnosis. We adjusted pack years to zero in patients that indicated they were never smokers or passive smokers. When pack years were provided as categories in the dataset, we used the mid‐points of the category. We categorized histological types according to the WHO Classification of Thoracic Tumors into adenocarcinoma (ACC), squamous‐cell carcinoma (SCC), large‐cell carcinoma (LCC), small‐cell carcinoma (SCLC), neuroendocrine tumors (including carcinoids and large‐cell neuroendocrine carcinomas, excluding SCLC) (NET), and other histology. The category other included patients with rare histological types such as adenosquamous carcinoma, sarcomatoid carcinomas, carcinosarcoma, salivary gland‐type tumors, and patients with unknown histological type. If a patient had no diagnosis of chronic pulmonary disease (COPD) documented in the dataset, we assumed they did not have a diagnosis of COPD. This might lead to an underestimation of patients selected for screening by the adapted PLCOm2012. We categorized patients' UICC stage using clinical and pathological TNM from the dataset. The edition of the UICC was provided with information on TNM and was used accordingly. When clinical and pathological TNM were both available, we used the pathological information rather than the clinical data.

The NLST, USPSTF, and DLCST selection criteria use quit times of <15 and <10 years for inclusion for screening, respectively. As quit time was not available in the dataset, we disregarded quit time when applying these screening criteria. This might lead to an overestimation of the sensitivity of these selection approaches, as some former smokers most probably had quit times greater than the thresholds set by the criteria. As some variables used in the calculation of the PLCOm2012noRace were not available in the dataset, we used an adapted version provided by the creator of the original PLCOm2012 model Martin Tammemägi. The original PLCOm2012 model uses the number of years smoked, and cigarettes smoked per day to measure the smoking intensity and includes a personal history of cancer and a family history of lung cancer, which were all missing in our dataset. The adapted version of the PLCOm2021 model included age, COPD, BMI, smoking status, and pack years. Using the adapted version of the PLCOm2021 made it possible to calculate the 6‐year risk for a larger proportion of the patients in the dataset improving the power of this analysis. As, other than in other variables with missing values, BMI was only missing in a small proportion of patients (12.5%) we used multiple imputations to fill in the missing values.

### 2.5 Comparison of characteristics

We compared the characteristics of patients selected by different selection criteria as well as between patients selected and not selected for screening. The reason for these comparisons was to determine differences between the criteria other than sensitivity as well as to detect areas for improvement of selection criteria in general.

### 2.6 Statistical analysis

Patient characteristics are presented as mean values with standard deviation (SD) for metric variables and absolute and relative frequencies for categorical variables. They were compared between included and excluded patients, and between selected and not selected patients using the Student's *t*‐test for metric variables, and Chi^2^‐test or fisher‐exact test, when cell numbers were <6, for categorical variables. Statistical significance for these comparisons was determined using two‐sided *p*‐values with alpha errors <0.05. Multiple imputations of BMI was performed using the R package *mice*, which uses conditional multiple imputations. Variables used in the imputation process were age, gender, and comorbidities COPD, asthma, cardiovascular disease (CVD), renal insufficiency, and diabetes mellitus. We calculated the sensitivity of the screening criteria as the proportion of patients selected for screening among the patients included in the analyses according to the exclusion criteria. We compared the sensitivity of the different criteria using the McNemar test for the comparison of proportions in dependent samples. To control the type I error rate we reduced the number of tests performed by limiting the comparison of the criteria to comparing the one with the best performance to all other criteria. Additionally, statistical significance was determined using two‐sided *p*‐values with Bonferroni‐adjusted alpha errors <0.00143 (0.05 divided by 35 tests). The precision of estimates was based on 99.857% confidence intervals (CI).

Data analysis was performed using R Version 4.0.0 and RStudio Version 1.4. Tables and figures were created in RStudio and Microsoft Excel.

## 3 RESULTS

### 3.1 Patient population and demographics

In total, 9481 patients with a thoracic malignancy were identified in the DZL data warehouse. Of these, 3588 had complete information on pack years and age and were included in the analysis. The mean age of the included patients included was 66.5 with an SD of 9.9 years and not significantly different compared to the excluded patients (66.2, SD = 10.0, *p*‐value = 0.19). Of all included patients 58.8% (*n* = 2106) were male compared to 58.6% (*n* = 3257, *p*‐value 0.90) of all excluded patients. BMI was available in 87.5% (*n* = 3141), and mean BMI was 26.1 with an SD of = 4.9. After imputation, mean BMI was 26.1 with the SD of 4.7. In excluded patients, BMI was 26.0 with an SD of 5.7 (*p*‐value = 0.44) and available in 18.9%. Stage at diagnosis was available in 99.2% (*n* = 3560) of patients and distributed as follows: in situ 0.2% (*n* = 8), stage I 21.3% (*n* = 760), stage II 12.9% (*n* = 460), stage III 31.4% (*n* = 1117), and stage IV 34.5% (*n* = 1229). Compared to excluded patients, stage was significantly different (stage I = 35.5%, stage II = 23.8%, stage III = 30.2%, stage IV = 10.4%, *p*‐value <0.0001), however, information was missing in excluded patients in 49% of patients. Histology was known for 99.7% of patients; 53.2% (*n* = 1904) of patients were diagnosed with adenocarcinoma, 25.5% (*n* = 913) had SCC, 9.7% (*n* = 346) SCLC, 5.1% (*n* = 181) NET, 0.6% LCC (*n* = 22), and 5.8% (*n* = 212) had a histology other than the aforementioned. The proportion of patients with adenocarcinoma, the major histologic subtype, was not significantly different in excluded patients (53.2%, *p*‐value 0.95). Smoking status was available in 99.8% of patients; 35.1% (*n* = 1259) indicated active smoking status, 51.8% (*n* = 1858) were former smokers, and 13.1% (471) were never smokers. Mean pack years were 47.3 with a SD of 21.3 years for active smokers and 37.7 with a SD of 23.9 years for former smokers. The proportion of patients with fewer than 30, 20, or 15 pack years was 48.3%, 35.0%, and 31.4% in females, which was higher compared to the proportion of males which were 26.2%, 17.1%, and 14.2%. Table 1 shows all patient characteristics, overall and stratified by gender.

**TABLE 1:** Characteristics of the study population.

Characteristics	Complete cases (*n* = 3588)		Male (*n* = 2106)		Female (*n* = 1478)	
	Mean	SD	Mean	SD	Mean	SD
Age at diagnosis	66.5	9.9	67.3	9.6	65.3	10.1
BMI	26.1	4.9	26.7	4.6	25.3	5.3
BMI (after imputation)	26.1	4.7	26.6	4.5	25.3	5.0
Pack years active smokers	47.3	21.3	51.9	22.6	40.9	17.3
Pack years ex‐smokers	37.7	23.9	41.8	25.1	30.2	19.2

	*n*	%	*n*	%	*n*	%
<50 years	160	4.5%	74	3.5%	86	5.8%
<55 years	376	10.5%	184	8.7%	192	13.0%
>80 years	234	6.5%	152	7.2%	82	5.5%
Male	2106	58.8%				
COPD	1120	31.2%	720	34.2%	400	27.1%
Asthma	47	1.3%	22	1.0%	25	1.7%
CVD	2329	64.9%	1467	69.7%	862	58.3%
Diabetes mellitus	611	17.0%	443	21.0%	168	11.4%
Renal insufficiency	188	5.2%	134	6.4%	54	3.7%
Stage at diagnosis						
0	8	0.2%	6	0.3%	2	0.1%
I	760	21.3%	420	20.0%	339	23.2%
II	460	12.9%	277	13.2%	182	12.4%
III	1117	31.4%	679	32.4%	438	30.0%
IV	1229	34.5%	719	34.3%	509	34.8%
Histology						
ACC	1904	53.2%	978	46.5%	924	62.8%
SCC	913	25.5%	692	32.9%	219	14.9%
SCLC	346	9.7%	203	9.7%	143	9.7%
LCC	22	0.6%	13	0.6%	9	0.6%
NET	181	5.1%	85	4.0%	96	6.5%
Other	212	5.8%	131	6.1%	81	5.4%
<30 pack years	1267	35.3%	552		714	48.3%
<20 pack years	879	24.5%	361	17.1%	517	35.0%
<15 pack years	765	21.3%	300	14.2%	464	31.4%
Smoking status						
Current	1259	35.1%	731	34.7%	528	35.7%
Former	1858	51.8%	1212	57.5%	642	43.4%
Never	471	13.1%	163	7.7%	308	20.8%

*Note*: Characteristics of study population (complete cases: age and smoking status available), stratified by gender. Means with standard deviation of numerical variables and absolute and relative frequency of categorical variables.

Abbreviations: ACC, adenocarcinoma; BMI, body mass index; COPD, chronic obstructive pulmonary disease; CVD, cardiovascular disease; LCC, large‐cell carcinoma; NET, neuroendocrine tumor; NSCLC, non‐small lung cancer; SCC, squamous‐cell carcinoma; SCLC, small‐cell carcinoma; SD, standard deviation.

### 3.2 Sensitivity of screening criteria to select patients for screening stratified by gender and histology

**FIGURE 1:**
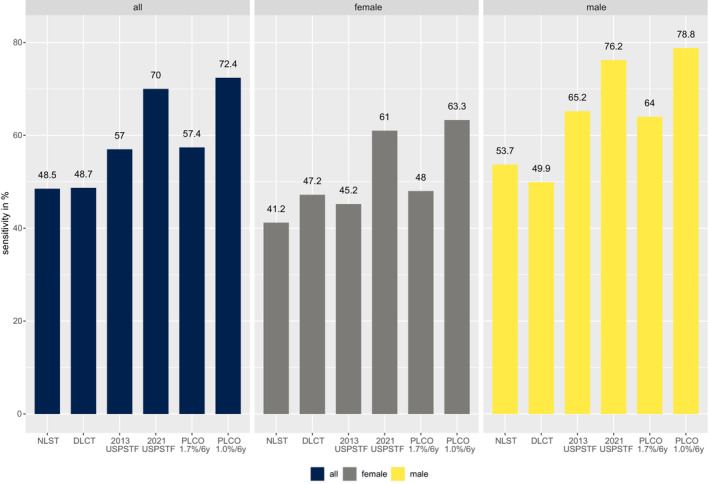
Sensitivity (%) of lung cancer selection criteria overall and stratified by gender. Sensitivity of lung cancer selection criteria to select patients diagnosed with lung cancer for screening. Stratification by gender. DLCT, Danish Lung Cancer Screening Trial; NLST, National Lung Screening Trial; PLCO, Prostate, Lung, Colon, and Ovarian risk prediction model; USPSTF, US Preventive Task Force.

Of all the lung cancer patients from the data warehouse included in the analysis, 72.4% (CI = 63.3% to 81.6%) were selected for screening by the adapted PLCOm2012 model with a risk threshold of 1.0%/6 years. This proportion was significantly higher compared to 70.0% (CI = 67.7% to 72.2%, *p*‐value = 0.001) in the 2021 USPSTF, 57.7% (CI = 55.0% to 59.9%, *p*‐value <0.0001) by the adapted PLCOm2012 with a threshold of 1.7%/6 years, 57.0% (CI = 54.5% to 59.5%, *p*‐value <0.0001) by the 2013 USPSTF, 48.7% (CI = 46.3% to 51.2%, *p*‐value <0.0001) by the DLCST, and 48.5% (CI = 46.0% to 51.0%, *p*‐value <0.0001) by the NLST. Among male patients, the sensitivity ranged from 78.8% (CI = 76.2% to 81.5%) using the adapted PLCOm2012 (1.0%/6 years) to 49.9% (CI = 46.6% to 53.1%, *p*‐value <0.0001) using the DLCST. The 2021 USPSTF selected 76.2% (CI = 73.4% to 79.0%, *p*‐value = 0.01) of patients, the 2013 USPSTF selected 65.2% (CI = 61.2% to 68.3%, *p*‐value <0.0001), the adapted PLCOm2012 (1.7%/6 years) selected 64.0% (CI = 60.9% to 67.1%, *p*‐value <0.0001), and the NLST had a sensitivity of 53.7% (CI = 50.4% to 56.9%, *p*‐value <0.0001). The sensitivity to select patients for screening in females was lower compared to males for all criteria. The sensitivity was highest when using the adapted PLCOm2012 with a threshold of 1.0%/6 years (63.3%, CI = 59.5% to 67.0%). The 2021 USPSTF criteria selected 61.0% (CI = 57.2% to 64.8%, *p*‐value = 0.02) of female patients, followed by 48.0% (CI = 44.2% to 51.9%, *p*‐value <0.0001) using the adapted PLCOm2012 with a threshold of 1.7%, 47.2% (CI = 43.4% to 51.1%, *p*‐value <0.0001) using the DLCST, 45.2% (CI = 41.3% to 49.1%, *p*‐value <0.0001) using the 2012 USPSTF, and 41.2% (CI = 37.4% to 45.0%, *p*‐value <0.0001) using the NLST criteria. Figure 1 displays these results.

Regarding histological types, the adapted PLCOm2012 with a threshold of 1.0%/6 years selected a higher proportion of patients for screening compared to all other selection criteria among all histological types, apart from SCLC and LCC where the USPSTF selected the highest proportion of patients (no statistical significance). Exact sensitivities with CI of all selection criteria among all histological types and stratified by sex, as well as *p*‐values can be found in Table 2.

**TABLE 2:** Sensitivities of selection criteria in % across histological type and gender.

All patients					Male				Female			
	*n*	%	CI	*p*‐value	*n*	%	CI	*p*‐value	*n*	%	CI	*p*‐value
	Adenocarcinoma (*n* = 1904)				Adenocarcinoma (*n* = 978)				Adenocarcinoma (*n* = 924)			
NLST	799	42.0%	[38.6%; 45.3]	<0.0001	452	46.2%	[41.5%; 51.0%]	<0.0001	346	37.4%	[32.7%; 42.2%]	<0.0001
DLCST	864	45.4%	[42.0%; 48.8%]	<0.0001	454	46.4%	[41.7%; 51.2%]	<0.0001	409	44.3%	[39.4%; 49.1%]	<0.0001
2013 USPSTF	919	48.3%	[44.8%; 51.7%]	<0.0001	544	55.6%	[50.9%; 60.4%]	<0.0001	374	40.5%	[35.7%; 45.3%]	<0.0001
2021 USPSTF	1181	62.0%	[58.7%; 65.4%]	0.006	666	68.1%	[63.7%; 72.5%]	0.003	514	55.6%	[50.8%; 60.5%]	0.42
PLCO 1.0%/6 years	1238	65.0%	[61.8%; 68.3%]	ref for *p*‐value	711	72.7%	[68.5%; 76.9%]	ref for *p*‐value	526	56.9%	[52.1%; 61.8%]	ref for *p*‐value
PLCO 1.7%/6 years	946	49.7%	[46.3%; 53.1%]	<0.0001	558	57.1%	[52.3%; 61.8%]	<0.0001	388	42.0%	[37.1%; 46.8%]	<0.0001

	SCC (*n* = 913)				SCC (*n* = 692)				SCC (*n* = 219)			
NLST	548	60.0%	[55.2%; 64.9%]	<0.0001	431	62.3%	[56.8%; 67.8%]	<0.0001	117	53.4%	[43.4%; 63.5%]	<0.0001
DLCST	491	53.8%	[48.9%; 58.7%]	<0.0001	377	54.5%	[48.8%; 60.1%]	<0.0001	114	52.1%	[42.0%; 62.1%]	<0.0001
2013 USPSTF	669	73.3%	[68.9%; 77.6%]	<0.0001	530	76.6%	[71.8%; 81.4%]	<0.0001	137	62.6%	[52.8%; 72.3%]	<0.0001
2021 USPSTF	776	85.0%	[81.5%; 88.5%]	0.27	600	86.7%	[82.9%; 90.6%]	0.79	174	79.5%	[71.3%; 87.6%]	0.09
PLCO 1.0%/6 years	791	86.6%	[83.3%; 90.0%]	ref for *p*‐value	604	87.3%	[83.5%; 91.1%]	ref for *p*‐value	185	84.5%	[77.2%; 91.8%]	ref for *p*‐value
PLCO 1.7%/6 years	663	72.6%	[68.2%; 77.0%]	<0.0001	509	73.6%	[68.6%; 78.6%]	<0.0001	152	69.4%	[60.1%; 78.7%]	<0.0001

	SCLC (*n* = 346)				SCLC (*n* = 203)				SCLC (*n* = 143)			
NLST	211	61.0%	[53.2%; 68.8%]	<0.0001	131	64.5%	[54.5%; 74.5%]	<0.0001	80	55.9%	[43.6%; 68.3%]	<0.0001
DLCST	216	62.4%	[54.7%; 70.2%]	<0.0001	122	60.1%	[49.8%; 70.3%]	<0.0001	94	65.7%	[53.9%; 77.6%]	0.003
2013 USPSTF	245	70.8%	[63.5%; 78.1%]	<0.0001	158	77.8%	[69.1%; 86.5%]	<0.0001	87	60.8%	[48.7%; 73.0%]	<0.0001
2021 USPSTF	297	85.8%	[80.2%; 91.4%]	ref for *p*‐value	180	88.7%	[82.0%; 95.3%]	ref for *p*‐value	117	81.8%	[72.2%; 91.4%]	1.00
PLCO 1.0%/6 years	289	83.5%	[77.6%; 89.5%]	0.39	172	84.7%	[77.2%; 92.3%]	0.23	117	81.8%	[72.2%; 91.4%]	ref for *p*‐value
PLCO 1.7%/6 years	239	69.1%	[69.1%; 76.5%]	<0.0001	146	71.9%	[62.5%; 81.3%]	<0.0001	93	65.0%	[53.1%; 76.9%]	<0.0001

	LCC (*n* = 22)				LCC (*n* = 13)				LCC (*n* = 9)			
NLST	12	54.5%	[22.9%; 86.2%]	<0.0001	7	53.8%	[12.6%; 95.1%]	0.22	5	55.6%	[8.8%; 95.3%]	0.48
DLCST	12	54.5%	[22.9%; 86.2%]	<0.0001	6	46.2%	[4.9%; 87.4%]	0.13	6	66.7%	[14.4%; 98.1%]	1.00
2013 USPSTF	15	68.2%	[38.6%; 97.8%]	<0.0001	9	69.2%	[22.9%; 96.9%]	0.62	6	66.7%	[14.4%; 98.1%]	1.00
2021 USPSTF	18	81.8%	[45.3%; 98.3%]	ref for *p*‐value	11	84.6%	[36.3%; 99.7%]	1.00	7	77.8%	[21.5%; 99.6%]	ref for *p*‐value
PLCO 1.0%/6 years	17	77.3%	[40.5%; 96.9%]	1.00	11	84.6%	[36.3%; 99.7%]	ref for *p*‐value	6	66.7%	[14.4%; 98.1%]	1.00
PLCO 1.7%/6 years	11	50.0%	[42.0%; 58.0%]	0.05	7	53.8%	[29.1%; 98.8%]	0.13	4	44.4%	[32.1%; 56.8%]	0.25

	NET (*n* = 181)				NET (*n* = 85)				NET (*n* = 96)			
NLST	64	35.4%	[24.8%; 46.0%]	<0.0001	39	45.9%	[29.8%; 62.0%]	0.02	25	26.0%	[12.7%; 39.4%]	0.001
DLCST	65	35.9%	[25.3%; 46.5%]	0.001	31	36.5%	[20.9%; 52.0%]	<0.0001	34	35.4%	[20.9%; 50.0%]	0.29
2013 USPSTF	69	38.1%	[27.4%; 48.9%]	0.0005	44	51.8%	[35.6%; 67.9%]	0.18	25	26.0%	[12.7%; 39.4%]	0.001
2021 USPSTF	86	47.5%	[36.4%; 58.6%]	0.58	46	54.1%	[38.0%; 70.2%]	0.42	40	41.7%	[26.7%; 56.7%]	1.00
PLCO 1.0%/6 years	90	49.7%	[38.6%; 60.8%]	ref for *p*‐value	50	58.8%	[42.9%; 74.7%]	ref for *p*‐value	40	41.7%	[26.7%; 56.7%]	ref for *p*‐value
PLCO 1.7%/6 years	71	39.2%	[28.4%; 50.1%]	<0.0001	40	47.1%	[30.9%; 63.2%]	0.004	31	32.3%	[18.1%; 46.5%]	0.008

	Others (*n* = 212)				Others (*n* = 131)				Others (*n* = 81)			
NLST	106	50.0%	[39.8%; 60.2%]	<0.0001	70	53.4%	[40.4%; 66.4%]	<0.0001	36	44.4%	[28.0%; 60.9%]	<0.0001
DLCST	101	47.6%	[37.4%; 57.9%]	<0.0001	60	45.8%	[32.8%; 58.8%]	<0.0001	41	50.6%	[34.0%; 67.2%]	0.000
2013 USPSTF	128	60.4%	[50.4%; 70.4%]	<0.0001	89	67.9%	[55.8%; 80.1%]	0.00	39	48.1%	[31.6%; 64.7%]	<0.0001
2021 USPSTF	152	71.7%	[62.5%; 80.9%]	0.003	102	77.9%	[67.0%; 88.7%]	0.08	50	61.7%	[45.6%; 77.8%]	0.02
PLCO 1.0%/6 years	173	81.6%	[73.7%; 89.5%]	ref for *p*‐value	112	85.5%	[76.3%; 94.7%]	ref for *p*‐value	61	75.3%	[49.7%; 81.2%]	ref for *p*‐value
PLCO 1.7%/6 years	130	61.3%	[51.3%; 71.3%]	<0.0001	88	67.2%	[54.9%; 79.4%]	<0.0001	41	50.6%	[34.0%; 67.2%]	<0.0001

*Note*: Sensitivity in % to detect lung cancer patients of different lung cancer screening criteria, stratified by histological type and gender. *p*‐values come from McNemar‐test and statistical significance determined using two‐sided *p*‐values with Bonferroni adjusted alpha errors <0.00143. Precision of estimates based on 99.857% confidence intervals (CI).

Abbreviations: DLCST, Danish Lung Cancer Screening Trial; LCC, large‐cell carcinoma; NET, neuroendocrine tumor; NLST, National Lung Screening Trial; PLCO, Prostate, Lung, Colon, and Ovarian risk prediction model; SCC, squamous‐cell carcinoma; SCLC, small‐cell carcinoma; USPSTF, US Preventive Task Force.

### 3.3 Comparison of patients selected by adapted PLCOm2012 and USPSTF

We compared the proportions of patients with specific characteristics (comorbidities, stage, and smoking status) selected by the adapted PLCOm2012 and USPSTF. The proportions of patients with comorbidities that were selected by the adapted PLCOm2012 were significantly higher than those selected by the USPSTF. It selected 6.9% more of the patients with COPD (92.5% vs. 85.6%, *p*‐value <0.0001), 5.3% more of the patients with CVD (76.7 vs. 71.4%, *p*‐value <0.0001), and 9.0% more of the patients with renal insufficiency (79.8% vs. 70.7%, *p*‐value = 0.01).

The adapted PLCOm2012 also performed significantly better in patients with stage I selecting 6.7% (70.8% vs. 64.1%, *p*‐value <0.0001) more patients compared to the USPSTF.

Additionally, the adapted PLCOm2012 selected 5.4% more current smokers (96.1% vs. 90.7%, *p*‐value <0.0001). It was also the only selection criteria to select never smokers for screening.

Table 3 displays the proportions of all characteristics, additionally stratified by gender.

**TABLE 3:** Comparison of proportions of patients with specific characteristics selected by the adapted PLCOm2012 and USPSTF.

	*n* total	All patients				diff	*p*‐value
		PLCO 1.0%		2021 USPSTF			
		*n*	%	*n*	%		
COPD	1120	1036	92.5%	959	85.6%	6.9%	<0.0001
Asthma	47	27	57.4%	25	53.2%	4.3%	0.75
CVD	2329	1786	76.7%	1662	71.4%	5.3%	<0.0001
Diabetes mellitus	611	474	77.6%	453	74.1%	3.4%	0.06
Renal insufficiency	188	150	79.8%	133	70.7%	9.0%	0.01
Stage at diagnosis							
0	8	5	62.5%	6	75.0%	−12.5%	1.00
I	760	538	70.8%	487	64.1%	6.7%	<0.0001
II	460	341	74.1%	340	73.9%	0.2%	1.00
III	1117	846	75.7%	828	74.1%	1.6%	0.27
IV	1229	864	70.3%	846	68.8%	1.5%	0.28
Smoking status							
Active	1259	1210	96.1%	1142	90.7%	5.4%	<0.0001
Former	1858	1357	73.0%	1368	73.6%	−0.6%	0.68
Never	471	31	6.6%	0	0.0%	6.6%	NA

	*n* total	Male				diff	*p*‐value
		PLCO 1.0%		2021 USPSTF			
		*n*	%	*n*	%		
*n*			1720		1605		
Mean age at diagnosis		69.0	8.1	66.8	7.4		
Male							
Mean BMI		26.6	4.4	26.7	5.3		
COPD	720	680	94.4%	624	86.7%	7.8%	<0.0001
Asthma	22	14	63.6%	15	68.2%	−4.5%	1.00
CVD	1467	1205	82.1%	1134	77.3%	4.8%	<0.0001
Diabetes mellitus	443	365	82.4%	355	80.1%	2.3%	0.31
Renal insufficiency	134	112	83.6%	102	76.1%	7.5%	0.09
Stage at diagnosis							
0	6	5	83.3%	5	83.3%	0.0%	NA
I	420	321	76.4%	281	66.9%	9.5%	<0.0001
II	277	228	82.3%	227	81.9%	0.4%	1.00
III	679	548	80.7%	552	81.3%	−0.6%	0.81
IV	719	556	77.3%	540	75.1%	2.2%	0.22
Smoking status							
Active	731	710	97.1%	672	91.9%	5.2%	<0.0001
Former	1212	944	77.9%	933	77.0%	0.9%	0.60
Never	163	6	0.0%	0	0.0%	0.0%	NA

	*n* total	Female				diff	*p*‐value
		PLCO 1.0%		2021 USPSTF			
		*n*	%	*n*	%		
*n*			915		902		
Mean age at diagnosis		67.1	7.6	64.8	7.1		
Male							
Mean BMI		25.0	4.9	25.2	5.2		
COPD	400	356	89.0%	335	83.8%	5.3%	0.4%
Asthma	25	13	52.0%	10	40.0%	12.0%	0.45
CVD	862	581	67.4%	528	61.3%	6.1%	<0.0001
Diabetes mellitus	168	109	64.9%	98	58.3%	6.5%	0.10
Renal insufficiency	54	38	70.4%	31	57.4%	13.0%	0.12
Stage at diagnosis							
0	2	0	0.0%	1	50.0%	−50.0%	NA
I	339	216	63.7%	205	60.5%	3.2%	0.21
II	182	113	62.1%	112	61.5%	0.5%	1.00
III	438	298	68.0%	276	63.0%	5.0%	0.02
IV	509	307	60.3%	306	60.1%	0.2%	1.00
Smoking status							
Active	528	500	94.7%	470	89.0%	5.7%	<0.0001
Former	642	410	63.9%	432	67.3%	−3.4%	0.16
Never	308	25	0.0%	0	0.0%	0.0%	NA

*Note*: Comparison of proportions of patients with specific characteristics (comorbidities, stage, smoking status) selected for screening by adapted PLCOm2012 with a threshold of 1% and by the 2021 USPSTF. Proportions are calculated to the base of all patients in each category. *p*‐values from McNemar test for paired samples.

Abbreviations: COPD, chronic obstructive pulmonary disease; CVD, cardiovascular disease, diff, difference.

### 3.4 Characteristics of selected and unselected patients

Compared to patients not selected for screening, patients selected by the adapted PLCOm2012 were significantly older (68.7 ± 8.2 vs. 60.6 ± 11.2 years, *p*‐value <0.0001). The prevalence of comorbidities such as COPD (39.9% vs. 8.5%, *p*‐value <0.0001), CVD (68.7% vs. 54.2%, *p*‐value <0.0001), diabetes mellitus (18.2% vs. 13.8%, *p*‐value = 0.002), and renal insufficiency (5.8% vs. 3.8%, *p*‐value = 0.02), was significantly higher than in patients not selected for screening. Other characteristics and results stratified by gender can be found in Table 4.

**TABLE 4:** Comparison of characteristics of patients selected and not selected by adapted PLCOm2012.

	Total				*p*‐value	Male				*p*‐value	Female				*p*‐value
	Selected		Not selected			Selected		Not selected			Selected		Not selected		
*n*		2598		990			1660		446			935		543	
Mean age at diagnosis	68.7	8.2	60.6	11.2	<0.0001	69.4	8.2	59.7	10.7	<0.0001	67.6	8.2	61.3	11.6	<0.0001
<50 years	17	0.7%	143	14.4%	<0.0001	10	0.6%	64	14.3%	<0.0001	7	0.7%	79	14.5%	<0.0001
<55 years	114	4.4%	262	26.5%	<0.0001	62	3.7%	122	27.4%	<0.0001	52	5.6%	140	25.8%	<0.0001
>80	200	7.7%	34	3.4%	<0.0001	138	8.3%	14	3.1%	0.0003	20	2.1%	62	11.4%	0.02
Male	1660	63.9%	446	45.1%	<0.0001										
COPD	1036	39.9%	84	8.5%	<0.0001	680	41.0%	40	9.0%	<0.0001	356	38.1%	44	8.1%	<0.0001
Asthma	27	1.0%	20	2.0%	0.03	14	0.8%	8	1.8%	0.11	13	1.4%	12	2.2%	0.33
CVD	1786	68.7%	543	54.8%	<0.0001	1205	72.6%	262	58.7%	<0.0001	581	62.1%	281	51.7%	0.0001
Diabetes mellitus	474	18.2%	137	13.8%	0.002	365	22.0%	78	17.5%	0.05	109	11.7%	59	10.9%	0.71
Renal insufficiency	150	5.8%	38	3.8%	0.02	112	6.7%	22	4.9%	0.20	38	4.1%	16	2.9%	0.34
Mean BMI	25.9	4.7	26.5	4.8	0.0004	26.5	4.5	27.2	4.2	0.003	24.9	5.0	26.0	5.1	<0.0001
Stage at diagnosis															
0	5	0.2%	3	0.3%	0.02	5	0.3%	1	0.2%	0.19	0	0.0%	2	0.4%	0.04
I	538	20.7%	222	22.4%		321	19.3%	99	22.2%		216	23.1%	123	22.7%	
II	341	13.1%	119	12.0%		228	13.7%	49	11.0%		113	12.1%	69	12.7%	
III	846	32.6%	271	27.4%		548	33.0%	131	29.4%		298	31.9%	140	25.8%	
IV	864	33.3%	365	36.9%		556	33.5%	163	36.5%		307	32.8%	202	37.2%	
Stage unavailable	4	0.2%	10	1.0%		2	0.1%	4	0.9%		1	0.1%	7	1.3%	
Histology															
ACC	1238	47.7%	666	67.3%	<0.0001	711	42.8%	267	59.9%	0.0005	526	56.3%	398	73.3%	0.005
SCC	791	30.4%	122	12.3%		604	36.4%	88	19.7%		185	19.8%	34	6.3%	
SCLC	289	11.1%	57	5.8%		172	10.4%	31	7.0%		117	12.5%	26	4.8%	
LCC	17	0.7%	5	0.5%		11	0.7%	2	0.4%		6	0.6%	3	0.6%	
NET	90	3.5%	91	9.2%		50	3.0%	35	7.8%		40	4.3%	56	10.3%	
Other	173	6.7%	49	4.9%		112	6.7%	23	5.2%		61	6.5%	26	4.8%	
Smoking status															
Active	1210	46.6%	49	4.9%	<0.0001	710	42.8%	21	4.7%	<0.0001	500	53.5%	28	5.2%	<0.0001
Former	1357	52.2%	501	50.6%		944	56.9%	268	60.1%		410	43.9%	232	42.7%	
Never	31	1.2%	440	44.4%		6	0.4%	157	35.2%		25	2.7%	283	52.1%	
<30 PY	526	20.2%	741	74.8%	<0.0001	265	16.0%	278	62.3%	<0.0001	208	22.2%	506	93.2%	<0.0001
<20 PY	258	9.9%	621	62.7%	<0.0001	129	7.8%	232	52.0%	<0.0001	128	13.7%	389	71.6%	<0.0001
<15 PY	190	7.3%	575	58.1%	<0.0001	87	5.2%	213	47.8%	<0.0001	260	27.8%	454	83.6%	<0.0001
Mean pack years active smokers	48.2	21.0	24.2	11.4	<0.0001	52.7	22.4	28.0	12.5	<0.0001	42.0	17.0	21.4	9.7	<0.0001
Mean pack years ex‐smokers	42.4	24.8	25.3	15.3	<0.0001	45.6	26.0	28.3	15.6	<0.0001	35.0	20.0	21.7	14.3	<0.0001

*Note*: Characteristics of patients selected and not selected for lung cancer screening using the adapted PLCOm2012 selection criterion, stratified by gender. Numerical variables are represented by means with standard deviation and categorical variables as absolute and relative frequencies. *p*‐values come from *t*‐test for numerical variables and chi^2^‐test and fisher exact test for categorical variables.

Abbreviations: ACC, adenocarcinoma; COPD, chronic obstructive pulmonary disease; CVD, cardiovascular disease; LCC, large‐cell carcinoma; NET, neuroendocrine tumor; SCC, squamous‐cell carcinoma; SCLC, small‐cell carcinoma.

Other than patients selected by the adapted PLCOm2012, patients selected by the 2021 USPSTF were significantly younger than patients not selected (66.1 SD 7.3 vs. 67.4 SD 14.1 years). However, this difference in age does not signify a clinically relevant difference and is probably due to the large sample size. Similar to the adapted PLCOm2012 the prevalence of the comorbidities COPD (38.2% vs. 14.9%, *p*‐value <0.0001), CVD (66.2% vs. 51.7%, *p*‐value = 0.01), and diabetes mellitus (18.0% vs. 14.7%, *p*‐value = 0.02), was significantly higher in patients selected by the USPSTF. There was no significant difference in renal insufficiency. The results of these comparisons, also stratified by gender, are presented in Table 5.

**TABLE 5:** Comparison of characteristics of patients selected and not selected by 2021 USPSTF.

	Total				*p*‐value	Male				*p*‐value	Female				*p*‐value
	Selected		Not selected			Selected		Not selected			Selected		Not selected		
*n*		2510		1078			1605		501			902		576	
Mean age at diagnosis	66.1	7.3	67.4	14.1	0.01	66.8	7.4	69.0	14.6	0.002	64.8	7.1	66.0	13.5	0.05
<50 years	0	0.0%	160	14.8%	<0.0001	0	0.0%	74	14.8%	<0.0001	0	0.0%	68	11.8%	<0.0001
<55 years	154	6.1%	222	20.6%	<0.0001	83	5.2%	101	20.2%	<0.0001	71	7.9%	121	21.0%	<0.0001
>80	0	0.0%	234	21.7%	<0.0001	0	0.0%	152	30.3%	<0.0001	0	0.0%	82	14.2%	<0.0001
Male	1605	63.9%	501	46.5%	<0.0001										
COPD	959	38.2%	161	14.9%	<0.0001	624	38.9%	96	19.2%	<0.0001	335	37.1%	65	11.3%	<0.0001
Asthma	25	1.0%	22	2.0%	0.02	15	0.9%	7	1.4%	0.52	10	1.1%	15	2.6%	0.05
CVD	1662	66.2%	557	51.7%	0.01	1134	70.7%	333	66.5%	0.08	528	58.5%	334	58.0%	0.88
Diabetes mellitus	453	18.0%	158	14.7%	0.02	355	22.1%	88	17.6%	0.03	98	10.9%	70	12.2%	0.50
Renal insufficiency	133	5.3%	55	5.1%	0.87	102	6.4%	32	6.4%	1.00	31	3.4%	23	4.0%	0.68
Mean BMI	26.2	5.3	26.7	12.9	0.18	26.7	5.3	26.9	3.9	0.48	25.2	5.2	26.6	17.3	0.06
Stage at diagnosis															
0	6	0.2%	2	0.2%	<0.0001	5	0.3%	1	0.2%	<0.0001	1	0.1%	1	0.2%	0.90
I	487	19.4%	273	25.3%		281	17.5%	139	27.7%		205	22.7%	134	23.3%	
II	340	13.5%	120	11.1%		227	14.1%	50	10.0%		112	12.4%	70	12.2%	
III	828	33.0%	289	26.8%		552	34.4%	127	25.3%		276	30.6%	162	28.1%	
IV	846	33.7%	383	35.5%		540	33.6%	179	35.7%		306	33.9%	203	35.2%	
Stage unavailable	3	0.1%	11	1.0%		0	0.0%	5	1.0%		2	0.2%	6	1.0%	
Histology															
ACC	1181	47.1%	723	67.1%	<0.0001	666	41.5%	312	62.3%	<0.0001	514	57.0%	410	71.2%	<0.0001
SCC	776	30.9%	137	12.7%		600	37.4%	92	18.4%		174	19.3%	45	7.8%	
SCLC	297	11.8%	49	4.5%		180	11.2%	23	4.6%		117	13.0%	26	4.5%	
LCC	18	0.7%	4	0.4%		11	0.7%	2	0.4%		7	0.8%	2	0.3%	
NET	86	3.4%	95	8.8%		46	2.9%	39	7.8%		40	4.4%	56	9.7%	
Other	152	6.1%	70	6.5%		102	6.4%	33	6.6%		50	5.5%	37	6.4%	
Smoking status												0		0.0%	
Active	1142	45.5%	117	10.9%	<0.0001	672	41.9%	59	11.8%	<0.0001	470	52.1%	58	10.1%	<0.0001
Former	1368	54.5%	490	45.5%		933	58.1%	279	55.7%		432	47.9%	210	36.5%	
Never	0	0.0%	471	43.7%		0	0.0%	163	32.5%		0	0.0%	308	53.5%	
<30 PY	346	13.8%	921	85.4%	<0.0001	167	10.4%	385	76.8%	<0.0001	179	19.8%	535	92.9%	<0.0001
<20 PY	0	0.0%	879	81.5%	<0.0001	0	0.0%	361	72.1%	<0.0001	0	0.0%	517	89.8%	<0.0001
<15 PY	0	0.0%	765	71.0%	<0.0001	0	0.0%	300	59.9%	<0.0001	0	0.0%	464	80.6%	<0.0001
mean pack years active smokers	49.1	20.4	29.7	21.5	<0.0001	53.2	22.1	38.0	24.0	<0.0001	43.3	16.1	21.2	14.4	<0.0001
mean pack years ex‐smokers	45.1	21.3	17.3	18.0	<0.0001	48.1	22.8	20.8	20.7	<0.0001	38.6	16.1	12.8	12.2	<0.0001

*Note*: Characteristics of patients selected and not selected for lung cancer screening using the 2021 USPSTF selection criterion, stratified by gender. Numerical variables are represented by means with standard deviation and categorical variables as absolute and relative frequencies. *p*‐values come from the *t*‐test for numerical variables and chi^2^‐test and fisher exact test for categorical variables.

Abbreviations: ACC, adenocarcinoma; COPD, chronic obstructive pulmonary disease; CVD, cardiovascular disease; LCC, large‐cell carcinoma; NET, neuroendocrine tumor; SCC, squamous‐cell carcinoma; SCLC, small‐cell carcinoma.

## 4 DISCUSSION

Given that the introduction of a national lung cancer screening program is planned in Germany, this study aimed to compare the performance of different screening algorithms to select lung cancer patients for screening. Using a cohort of lung cancer patients documented as part of a research consortium, we were able to compare the sensitivity of the selection criteria. We found that out of the screening algorithms compared in this study the adapted PLCOm2012 model with a threshold of 1.0%/6 years outperformed the other screening criteria, mostly regardless of histologic type and gender of the lung cancer patients. The 2021 USPSTF criterion had the second‐highest sensitivity. The sensitivity of the adapted PLCOm2022 at both thresholds in this sample was lower than observed in other populations in which predictor variable data were more complete and the original model was used, suggesting that the sensitivities in this study may be underestimates of what can be expected when this model would be applied in the German population in practice, where complete accurate data are collected.

In all analyses, we found that all selection criteria performed worse in females than in males. However, while in males the sensitivity of the adapted PLCOm2012 was 81.8% compared to 76.2% with the USPSTF, the difference in females was only marginal (63.3% vs. 61.0%). Pasquinelli et al. reported that women diagnosed with lung cancer typically have less of a smoking history and are less likely to be eligible by the 2013 USPSTF criteria compared to men and that expanding eligibility criteria (such as in the 2021 USPSTF) would decrease but not eliminate this gap.^18^ This gender disparity is present in our analysis of German data. A single‐center retrospective study similar to ours in the United States of America found that of 294 female lung cancer patients, only 19.4% would have been selected for screening by the 2013 USPSTF criteria,^19^ which is even lower than the 45.2% we found in this study. An interesting additional finding of Vu et al. was that 48.1% of females not selected for screening by the USPSTF had a family history of cancer, which is a criterion included in the original PLCO risk model. A study across several countries in Europe comparing the sensitivity and specificity of smoking‐based screening criteria found similar trends with regard to gender. The sensitivity in men than in women was higher in general, as well as in the German cohort. However, the specificity was higher in women.^20^ Therefore, the loss in specificity when expanding selection criteria for females to increase sensitivity might still be reasonable.

All selection criteria performed better in histological types associated with a higher attributable risk of smoking, such as SCC and SCLC. This finding is not surprising as smoking history is one of the two main factors incorporated in the selection criteria. However, as adenocarcinomas represent the highest proportion of lung cancers and have a lower attributable risk of smoking, this aspect underlines the need to incorporate factors other than smoking and age into screening algorithms. In addition, the proportion of women with adenocarcinomas is higher, which maybe one of the reasons for the lower sensitivity of selection criteria compared with that of males. Given the rising lung cancer incidence in females and the fact that the incidence in males is plateauing, a more gender‐specific approach to screening is expected to benefit screening programs. One approach to this, which is available to model‐based approaches, is to add a specific predictor term for gender, which increased risk in women. Two existing models, the Bach and LCRAT/LCDRAT include predictor terms for gender but are counterproductive regarding gender disparity because they reduce the risk for women and lower the probability of selection of women for screening.

Disregarding model‐based selection criteria, an important finding of this study was, that the sensitivity of the 2013 USPSTF criteria was higher than that of the NLST for the whole sample and also in all subgroups. The only difference between these criteria is the upper age of 75 versus 80 years, meaning the difference in sensitivities is attributable to upper age differences. This shows how the choice of selection criteria and small differences in eligibility criteria can have an impact on public health practice and possibly cost‐effectiveness.

When comparing other characteristics of patients selected by USPFTF and the adapted PLCOm2012, we found that the adapted PLCOm2012 selected a significantly higher proportion of the patients with stage I disease at diagnosis. This is important in terms of life years gained which is a factor that is considered when analyzing the cost‐effectiveness of screening programs. The two largest trials of lung cancer screening the NLST^1^ and the NELSON^21^ trial reported a stage shift when implementing lung cancer screening programs, and numerous follow‐up studies and systematic reviews support these findings.^22^, ^23^

The adapted PLCOm2012 resulted in the selection of a patient cohort with a significantly higher comorbidity burden compared to patients not selected for screening. For example, in total, 5.2% of patients in the dataset had renal insufficiency. The proportion of those patients selected by the adapted PLCOm2012 was higher (+9.0%) compared to the USPTF and represented almost 80% of all patients in this subgroup. It will be important to consider comorbidities when planning the follow‐up of detected lesions and the treatment of detected cancers.

A limitation of our analysis derives from the nature of datasets within the DZL data warehouse. The DZL data warehouse is a multicenter data pool based on data from five academic lung cancer centers in Germany, and, as such, might not perfectly represent the general population of lung cancer patients in Germany. For instance, there may be socioeconomic differences between patients treated at academic centers and the broader population. In addition, within the DZL various contributing departments may provide different types of clinical data, leading to the missing stage and smoking data in some datasets. Due to the strong research focus of thoracic surgery departments within the DZL, early‐stage lung cancer may be overrepresented in our cohort. However, this may provide more insight into patients with lower stage who in fact are the focus of lung cancer screening programs. Due to incomplete documentation of some basic clinical parameters in datasets from some contributing researchers, we had to exclude a large number of patients due to missing age at diagnosis or smoking status. When comparing included and excluded patients from the data warehouse we did not find significant differences concerning age, gender, and BMI. Stage at diagnosis was significantly different in the included dataset; however, the cohort of patients included in the analysis was a better reflection of the expected distribution of disease stage compared to the excluded patients. We suspect that patients excluded due to incomplete data may have had missing information on distant metastases, and may have had stage IV disease. Unfortunately, due to the high number of data sets with missing information on smoking history, we had to exclude a large number of patients with stage I and II disease who would have added substantial power to our analysis. We encourage public and academic data repositories to emphasize complete documentation of a basic clinical dataset including lung cancer risk factors such as smoking.

Within the cohort used for the analysis, some variables used in the calculation of the PLCOm2012 were not available in the dataset or were available but in a different format. Therefore, we used an adapted model of the PLCOm2012. Although this model did include fewer variables the AUC was still very high (0.8375). Overall, using this adapted model might lead to an underestimation of the sensitivity of the PLCOm2012, so conclusions regarding the PLCOm2012 are conservative. Another limitation of the analysis is that due to the nature of the dataset and lack of inclusion of at‐risk individuals without lung cancer, we were not able to compare the specificity of the screening criteria. However, with regard to comparisons between USPSTF and PLCOm2012, risk thresholds were selected which have been shown in other studies to yield similar numbers being found to be eligible by both criteria, thus allowing for fair comparisons.

One of the strengths of this study was the large size of the dataset, which allowed subset analyses. Many other analyses comparing screening criteria have been limited to lung cancer screening studies, in which few lung cancer cases were available, and subset analyses by histological subtypes were constrained. Furthermore, our analysis included patients from all stages with differing treatment indications. Additionally, even though, one goal of lung cancer screening is to detect patients in early stages we believe that also including patients with higher stages can help to detect areas for improvement of selection criteria in general to improve their sensitivity also in these stages. Additionally, using this retrospective approach, our results provide the important aspect of real‐world evidence.

## 5 CONCLUSION

Using a risk‐based selection approach resulted in higher sensitivities compared to criteria using dichotomized categorical age and smoking history in a German population of lung cancer patients. However, gender disparities were apparent in all studied eligibility criteria. In light of increasing lung cancer incidences in women, all selection criteria should be reviewed regarding ways to close this gender gap, especially when implementing a large‐scale lung cancer screening program.

## AUTHOR CONTRIBUTIONS

**Julia Walter:** Conceptualization (equal); formal analysis (lead); investigation (lead); methodology (lead); project administration (equal); writing – original draft (lead); writing – review and editing (lead). **Diego Kauffmann‐Guerrero:** Investigation (supporting); supervision (supporting); writing – original draft (supporting); writing – review and editing (supporting). **Thomas Muley:** Data curation (equal); methodology (supporting); writing – original draft (supporting). **Martin Reck:** Data curation (equal); methodology (supporting); writing – original draft (supporting). **Jan Fuge:** Data curation (equal); formal analysis (supporting); writing – original draft (supporting). **Andreas Günther:** Data curation (equal); writing – original draft (supporting). **Raphael W. Majeed:** Data curation (lead); validation (equal); writing – original draft (supporting). **Rajkumar Savai:** Data curation (equal); writing – original draft (supporting). **Ina Koch:** Data curation (equal); writing – original draft (supporting). **Julien Dinkel:** Data curation (equal); writing – original draft (supporting). **Christian Schneider:** Data curation (equal); writing – original draft (supporting). **Karsten Senghas:** Data curation (equal); writing – original draft (supporting). **Sonja Kobinger:** Data curation (equal); writing – original draft (supporting). **Farkhad Manapov:** Data curation (equal); writing – original draft (supporting). **Michael Thomas:** Data curation (equal); writing – original draft (supporting). **Kathrin Kahnert:** Data curation (equal); writing – original draft (supporting). **Hauke Winter:** Data curation (equal); writing – original draft (supporting). **Jürgen Behr:** Data curation (equal); supervision (supporting); writing – original draft (supporting). **Martin Tammemägi:** Data curation (equal); methodology (equal); writing – original draft (supporting). **Amanda Tufman:** Data curation (equal); methodology (supporting); project administration (supporting); supervision (lead); writing – original draft (supporting); writing – review and editing (supporting).

## FUNDING INFORMATION

TM, KS, SK, MT, and HW: Supported in part by the German Federal Ministry of Education and Research grants 82DZL00402, 82DZL004A2 and 82DZL004B2. IK: Supported in part by the German Federal Ministry of Education and Research grants 82DZL00303, 82DZL003A3 and 82DZL003B3.

## CONFLICT OF INTEREST STATEMENT

The authors whose names are listed immediately below certify that they have NO affiliations with or involvement in any organization or entity with any financial interest (such as honoraria; educational grants; participation in speakers' bureaus; membership, employment, consultancies, stock ownership, or other equity interest; and expert testimony or patent‐licensing arrangements), or non‐financial interest (such as personal or professional relationships, affiliations, knowledge or beliefs) in the subject matter or materials discussed in this manuscript.

## ETHICS STATEMENT

Approval for this retrospective non‐interventional study was obtained from the Ethics Committee of the Ludwig‐Maximilians University (reference number 19–959). This study was conducted in accordance with the Declaration of Helsinki, Good Clinical Practice guidelines, and local ethical and legal requirements.

## CONSENT FOR PUBLICATION

Not applicable.

## Supporting information


Table S1

## Data Availability

The data that support the findings of this study on request from the corresponding author with the permission of DZL (German Center for Lung Research). Restrictions apply to the availability of these data.
