# Supplementary material for: Comparison of the sensitivity of different criteria to select lung cancer patients for screening in a cohort of German patients
Source: Cancer Med. 2023 Jan 27;12(7):8880–96. doi: 10.1002/cam4.5638 (PMC10134298; doi:10.1002/cam4.5638)
Supplement: Supplementary file 1 — Table S1 [file CAM4-12-8880-s001.docx]

**Supplemental table 1**

|  | complete cases  (n = 3588) | | |  | excluded cases (n = 5849) | | | p-value |
| --- | --- | --- | --- | --- | --- | --- | --- | --- |
|  | n missing | mean | sd |  | n missing | mean | sd |  |
| age at diagnosis | 0 | 66.5 | 9.9 |  | 1880 | 66.2 | 10.0 | 0.19 |
| BMI | 447 | 26.3 | 8.9 |  | 4745 | 26.0 | 5.7 | 0.44 |
|  |  | n | % |  |  |  |  |  |
| sex | 4 |  |  |  | 291 |  |  |  |
| male |  | 2106 | 58.8% |  |  | 3257 | 58.6% |  |
| female |  | 1478 | 41.2% |  |  | 2301 | 39.3% | 0.90 |
| comorbidities |  |  |  |  |  |  |  |  |
| COPD |  | 1120 | 31.2% |  |  | 560 | 9.6% | <0.0001 |
| asthma |  | 47 | 1.3% |  |  | 23 | 0.4% | <0.0001 |
| CVD |  | 2329 | 64.9% |  |  | 1322 | 22.6% | <0.0001 |
| diabetes mellitus |  | 611 | 17.0% |  |  | 332 | 5.7% | <0.0001 |
| renal isufficiency |  | 188 | 5.2% |  |  | 70 | 1.2% | <0.0001 |
| stage at diagnosis | 14 |  |  |  | 2883 |  |  |  |
| 0 |  | 8 | 0.2% |  |  | 4 | 0.1% | <0.0001 |
| I |  | 760 | 21.3% |  |  | 1053 | 35.5% |  |
| II |  | 460 | 12.9% |  |  | 705 | 23.8% |  |
| III |  | 1117 | 31.4% |  |  | 896 | 30.2% |  |
| IV |  | 1229 | 34.5% |  |  | 308 | 10.4% |  |
| histology | 11 |  |  |  | 834 |  |  |  |
| ACC |  | 1904 | 53.2% |  |  | 2666 | 53.2% | 0.95 |
| SCC |  | 913 | 25.5% |  |  | 1449 | 28.9% | 0.001 |
| SCLC |  | 346 | 9.7% |  |  | 183 | 3.6% | <0.0001 |
| LCC |  | 22 | 0.6% |  |  | 98 | 2.0% | <0.0001 |
| NET |  | 181 | 5.1% |  |  | 394 | 7.9% | <0.0001 |
| other |  | 211 | 5.9% |  |  | 225 | 4.5% | 0.004 |
| missing |  | 11 | 0.3% |  |  | 834 | 14.3% |  |

Comparison of characteristics of included and excluded patients. Means with standard deviation of numerical variables and absolute and relative frequency of categorical variables. Comparison of metric variables using Students t-test and categorical variables using Chi^2^-test.

SD = standard deviation, BMI = body mass index, COPD = chronic obstructive pulmonary disease, CVD = cardiovascular disease, ACC = adenocarcinoma, SCC = squamous-cell carcinoma, SCLC = small-cell carcinoma, LCC = large-cell carcinoma, NET = neuroendocrine tumor.
